# Respiratory electron transfer pathways in plant mitochondria

**DOI:** 10.3389/fpls.2014.00163

**Published:** 2014-04-29

**Authors:** Peter Schertl, Hans-Peter Braun

**Affiliations:** Abteilung Pflanzenproteomik, Institut für Pflanzengenetik, Leibniz Universität HannoverHannover, Germany

**Keywords:** plant mitochondria, electron transport chain, dehydrogenase, alternative oxidase, respiratory supercomplex

## Abstract

The respiratory electron transport chain (ETC) couples electron transfer from organic substrates onto molecular oxygen with proton translocation across the inner mitochondrial membrane. The resulting proton gradient is used by the ATP synthase complex for ATP formation. In plants, the ETC is especially intricate. Besides the “classical” oxidoreductase complexes (complex I–IV) and the mobile electron transporters cytochrome c and ubiquinone, it comprises numerous “alternative oxidoreductases.” Furthermore, several dehydrogenases localized in the mitochondrial matrix and the mitochondrial intermembrane space directly or indirectly provide electrons for the ETC. Entry of electrons into the system occurs via numerous pathways which are dynamically regulated in response to the metabolic state of a plant cell as well as environmental factors. This mini review aims to summarize recent findings on respiratory electron transfer pathways in plants and on the involved components and supramolecular assemblies.

## Introduction

During cellular respiration, organic compounds are oxidized to generate usable chemical energy in the form of ATP. The respiratory electron transport chain (ETC) of mitochondria is at the center of this process. Its core consists of four oxidoreductase complexes, the NADH dehydrogenase (complex I), the succinate dehydrogenase (complex II), the cytochrome c reductase (complex III) and the cytochrome c oxidase (complex IV), as well as of two mobile electron transporters, cytochrome c, and the lipid ubiquinone. Overall, electrons are transferred from the coenzymes NADH or FADH_2_ onto molecular oxygen which is reduced to water. Three of the four oxidoreductase complexes (complexes I, III and IV) couple their electron transfer reactions with proton translocation across the inner mitochondrial membrane. As a result, a proton gradient is formed which can be used by the ATP synthase complex (complex V) for the phosphorylation of ADP. In its classically described form, cellular respiration is based on a linear ETC (from NADH via complexes I, III, and IV to molecular oxygen). However, electrons can enter and leave the ETC at several alternative points. This is especially true for the plant ETC system, which is highly branched. In this review we aim to integrate current knowledge on the ETC system in plants with respect to its components, electron transport pathways and supramolecular structure.

## Components of the plant ETC system

**Figure 1:**
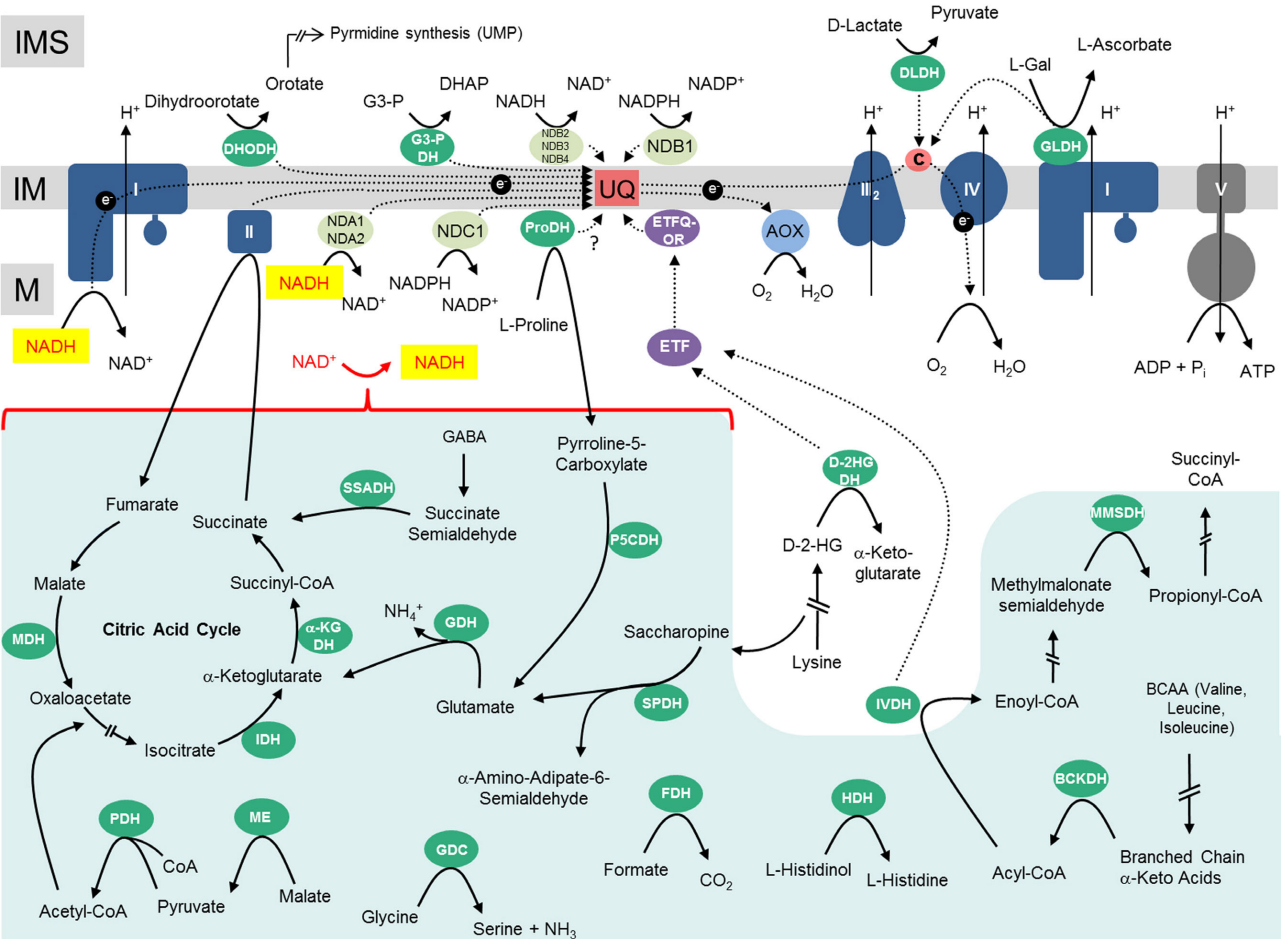
**Mitochondrial dehydrogenases and the respiratory chain**. Within the mitochondrial matrix (M) numerous dehydrogenases generate NADH by oxidizing various carbon compounds. NADH subsequently is re-oxidized at the inner mitochondrial membrane (IM) by the respiratory electron transfer chain (ETC). The electrons of NADH can enter the ETC through complex I or at the ubiquinone level via alternative NAD(P)H-dehydrogenases. Besides, some dehydrogenases of the mitochondrial matrix transfer electrons to ubiquinone via the ETF/ETFQOR system. Proline dehydrogenase possibly directly transfers electrons onto ubiquinone. In the intermembrane space (IMS), electrons from NAD(P)H generated in the cytoplasm can be inserted into the ETC via alternative NAD(P)H dehydrogenases. Furthermore, some dehydrogenases of the IMS can directly transfer electrons onto ubiquinone or cytochrome c. Color code—dark blue, protein complexes of the ETC; blue, AOX; purple, ETF/ETFQQ system; light green, alternative NAD(P)H dehydrogenases of the ETC; green, dehydrogenases; red, ubiquinone and cytochrome c; yellow, NADH produced by dehydrogenases of the mitochondrial matrix/NADH re-oxidized by complex I or internal alternative NADH dehydrogenases; dark gray, ATP synthase complex; light green background, NADH producing dehydrogenases of the mitochondrial matrix. Abbreviations—*alphabetically ordered*. I, complex I; II, complex II; III, complex III; IV, complex IV; V, complex V; α-KGDH, α-ketoglutarate dehydrogenase; AOX, alternative oxidase; BCKDH, branched-chain α-ketoacid dehydrogenase complex; c, cytochrome c; D-2HGDH, D-2-hydroxyglutarate dehydrogenase; DHODH, dihydroorotate dehydrogenase; DLDH, D-lactate dehydrogenase; ETF, electron transfer flavoprotein; ETFQOR, electron transfer flavoprotein ubiquinone oxidoreductase; FDH, formate dehydrogenase; GDC, glycine dehydrogenase; GDH, glutamate dehydrogenase; GLDH, L-galactono-1,4-lactone dehydrogenase; G3-PDH, glyceraldehyde 3-phosphate dehydrogenase; HDH, histidinol dehydrogenase; IDH, isocitrate dehydrogenase; IVDH, isovaleryl-coenzyme A dehydrogenase; MDH, malate dehydrogenase; ME, malic enzyme; MMSDH, methylmalonate-semialdehyde dehydrogenase; NDA1/2, NDB2/3/4, alternative NADH dehydrogenase; NDC1, NDB1, alternative NADPH dehydrogenase; P5CDH, pyrroline-5-carboxylate dehydrogenase; PDH, pyruvate dehydrogenase; ProDH, proline dehydrogenase; SPDH, saccharopine dehydrogenase; SSADH, succinic semialdehyde dehydrogenase; UQ, ubiquinone. For further information of the enzymes see Table 1.

The “classical” oxidoreductase complexes of the respiratory chain (given in dark blue in Figure 1) resemble their homologues in animal mitochondria but at the same time have some clear distinctive features (reviewed in Millar et al., 2008, 2011; Rasmusson and Moller, 2011; van Dongen et al., 2011; Jacoby et al., 2012). *Complex I* is especially large in plant mitochondria and includes nearly 50 different subunits (Braun et al., 2014). Compared to its homologs from bacteria and other eukaryotic lineages it has an extra domain which includes carbonic anhydrase-like proteins. The function of this additional domain is currently unclear but it has been suggested to be important in the context of an inner-cellular CO_2_ transfer mechanism to provide mitochondrial CO_2_ for carbon fixation in chloroplasts (Braun and Zabaleta, 2007; Zabaleta et al., 2012). *Complex II* is composed of four subunits in bacteria and mitochondria of animals and fungi. In plants complex II includes homologs of these subunits but additionally four extra proteins of unknown function (Millar et al., 2004; Huang and Millar, 2013). In contrast, the subunit composition of *complex III* from plants is highly similar to the ones in yeast and bovine mitochondria (Braun and Schmitz, 1995a). The two largest subunits of this protein complex, termed “core proteins” in animals and fungi, are homologous to the two subunits of the mitochondrial processing peptidase (MPP) which removes pre-sequences of nuclear-encoded mitochondrial proteins after their import into mitochondria. In animal mitochondria, the core proteins are proteolytically inactive. Instead, an active MPP is present within the mitochondrial matrix. In contrast, the core subunits of complex III from plants have intact active sites (Braun et al., 1992; Glaser et al., 1994). Indeed, complex III isolated from plant mitochondria efficiently removes pre-sequences of mitochondrial pre-proteins. The differing functional states of complex III in diverse eukaryotic lineages might reflect different evolutionary stages of this protein complex (Braun and Schmitz, 1995b). Also *complex IV* has some extra subunits in mitochondria of plants (Millar et al., 2004). Eight subunits are homologous to complex IV subunits from other groups of eukaryotes and another six putative subunits represent proteins of unknown functions.

The ETC of plant mitochondria additionally includes several so-called “alternative oxidoreductases”: the alternative oxidase (AOX; light blue in Figure 1) and several functionally distinguishable alternative NAD(P)H dehydrogenases (alternative NDs, light green in Figure 1). Findings on their functional roles have been reviewed recently (Rasmusson et al., 2008; Rasmusson and Moller, 2011; Moore et al., 2013). AOX directly transfers electrons from ubiquinol to molecular oxygen and therefore constitutes an alternative electron exit point of the ETC. As a result, complexes III and IV are excluded from respiratory electron transport. The alternative NAD(P)H dehydrogenases serve as alternative electron entry points of the plant ETC and may substitute complex I. They differ with respect to co-factor requirement and localization at the outer or inner surface of the inner mitochondrial membrane (external alternative NDs, internal alternative NDs). Some of the genes encoding alternative NDs are activated by light (Rasmusson et al., 2008; Rasmusson and Moller, 2011). The latter enzymes are considered to be important during photorespiration and all alternative enzymes during various stress conditions. Since none of the alternative oxidoreductases couple electron transfer with proton translocation across the inner mitochondrial membrane, their enzymatic function is believed to be important in the context of an overflow protection mechanism for the ETC which is especially relevant during high-light conditions.

Finally, dehydrogenases (dark green in Figure 1; Table 1) can directly or indirectly insert electrons into the respiratory chain (Rasmusson et al., 2008; Rasmusson and Moller, 2011). Numerous dehydrogenases of the mitochondrial matrix generate NADH which is re-oxidized by complex I and the internal alternative NDs. However, some dehydrogenases directly transfer electrons onto ubiquinone [dihydroorotate dehydrogenase (DHODH), glyceraldehyde 3-phosphate dehydrogenase (G3-PDH) and possibly proline dehydrogenase (ProDH)] or onto cytochrome c [L-galactone-1,4-lactone dehydrogenase (GLDH) and D-lactate dehydrogenase (DLDH)]. Furthermore, at least two dehydrogenases [isovaleryl-coenzyme A dehydrogenase (IVDH) and D-2-hydroxyglutarate dehydrogenase (D-2HGDH)] transfer electrons onto ubiquinone via a short electron transfer chain composed of the “electron transfer flavoprotein” and the “electron transfer flavoprotein-ubiquinone oxidoreductase” (ETF and ETFQ-OR, purple in Figure 1) (Ishizaki et al., 2005, 2006; Araújo et al., 2010). IVDH is involved in the branched chain amino acid catabolism and D-2HGDH in the catabolism of lysine. In plants, degradation of amino acids for respiration was shown to be especially important during carbon starvation conditions, e.g., extended darkness (Araújo et al., 2011). In contrast to animal mitochondria, fatty acid oxidation does not take place in plant mitochondria and the involved dehydrogenases consequently are absent. Instead, additional metabolic pathways occur in plants, e.g., the final step of an ascorbic acid biosynthesis pathway, which is catalyzed by GLDH. Electrons of L-galactono-1,4-lactone (GL) oxidation are inserted into the ETC via cytochrome c (Bartoli et al., 2000). Proline, besides being a building block for protein biosynthesis, is used as an osmolyte in plant cells. Proline is catabolized in mitochondria by a two-step process involving pyrroline-5-carboxylate dehydrogenase (P5CDH) and ProDH (Szabados and Savouré, 2010). P5CDH produces NADH, whereas ProDH represents a flavoenzyme which is assumed to transfer electrons directly or indirectly onto ubiquinone. Some additional dehydrogenases occur in plant mitochondria in the mitochondrial matrix and the intermembrane space which also contribute electrons to the ETC (Figure 1, Table 1). However, in some cases their mitochondrial localization is not entirely certain and should be further investigated by future research.

**Table 1:** **Mitochondrial dehydrogenases in *Arabidopsis thaliana*^a^**.

**Enzyme**	**Accession no.^b^** subunits isoforms etc.	**Catalysed reaction**	**Oligomeric state** Native mass/monomer mass according to GelMap^c^ (according to other data in the literature)	**Publication^d^** for Arabidopsis (for other plants)
Malate dehydrogenase	At1g53240	Malate + NAD^+^ ⇔ Oxaloacetate + NADH	At1g53240: 89 kDa/42 kDa	Journet et al., 1981
	At3g15020		At3g47520: 157 kDa/38 kDa	Gietl, 1992
				Krömer, 1995
				Nunes-Nesi et al., 2005
				Lee et al., 2008
				Tomaz et al., 2010
Isocitrate dehydrogenase	At4g35260	Isocitrate + NAD^+^ ⇔ α-Ketoglutarate + CO_2_ + NADH	At4g35260: 89 kDa/42 kDa	Behal and Oliver, 1998
	At5g14590		At5g14590: 140 kDa/53 kDa	Lancien et al., 1998
	At4g35650		At3g09810: 138 kDa/40 kDa	Lin et al., 2004
	At3g09810		At5g03290: 138 kDa/40 kDa	Lemaitre and Hodges, 2006
	At5g03290			Lemaitre et al., 2007
	At2g17130			
α-Ketoglutarate dehydrogenase complex	At3g55410 (E1)	α-Ketoglutarate + coenzyme A + NAD^+^ ⇔ succinyl-CoA + CO_2_ + NADH	At5g65750: 207 kDa/91 kDa	Poulsen and Wedding, 1970
	At5g65750 (E1)		At3g55410: 207 kDa/91 kDa	Wedding and Black, 1971a,b
	At4g26910 (E2)			Dry and Wiskich, 1987
	At5g55070 (E2)		(1.7 MDa complex)	Millar et al., 1999
	At3g17240 (E3)			Araújo et al., 2008
	At1g48030 (E3)			Araújo et al., 2013
	At3g13930 (E3)			
Glutamate dehydrogenase	At5g18170	Glutamate + H_2_O + NAD^+^ ⇔ α-Ketoglutarate + NH^+^_4_ + NADH	At5g18170: 209 kDa/48 kDa	Yamaya et al., 1984
	At5g07440		At5g07440: 209 kDa/48 kDa	Turano et al., 1997
	At3g03910		At3g03910: 209 kDa/48 kDa	Aubert et al., 2001
				Miyashita and Good, 2008a,b
				Fontaine et al., 2012
				Tarasenko et al., 2013
				Fontaine et al., 2012
Malic enzyme	At2g13560	Malate + NAD^+^ ⇔ Pyruvate + NADH + CO_2_	At2g13560: 370 kDa/63 kDa	Jenner et al., 2001
	At4g00570		At4g00570: 370 kDa/63 kDa	Tronconi et al., 2008
	At1g79750			Tronconi et al., 2010
				Tronconi et al., 2012
Pyruvate dehydrogenase complex	At1g59900 (E1)	Pyruvate + coenzyme A + NAD^+^ ⇔ Acetyl-CoA + CO_2_ + NADH	At3g13930: 1500 kDa/54 kDa	Luethy et al., 1994
	At1g24180 (E1)		At1g24180: 470 kDa/41 kDa	Grof et al., 1995
	At5g50850 (E1)		At5g50850: 150 kDa/39 kDa	Zou et al., 1999
	At3g52200 (E2)		At1g59900: 138 kDa/44 kDa	Tovar-Méndez et al., 2003
	At1g54220 (E2)			Szurmak et al., 2003
	At3g13930 (E3)		(9.5 MDa complex)	Yu et al., 2012
	At3g17240 (E3)			
	At1g48030 (E3)			
Glycine dehydrogenase complex	At4g33010 (P)	Glycine + H_4_ folate + NAD^+^ ⇔ methylene-H_4_ folate + CO_2_ + NH_3_ + NADH	At4g33010: 144 kDa/91 kDa	Somerville and Ogren, 1982
	At2g26080 (P)		At2g26080: 209 kDa/91 kDa	Oliver et al., 1990
	At1g32470 (H)		At1g11860: 148 kDa/46 kDa	Oliver, 1994
	At2g35120 (H)			Srinivasan and Oliver, 1995
	At2g35370 (H)		(1.3 MDa complex)	Douce et al., 2001
	At1g11860 (T)			
	At4g12130 (T)			
	At3g17240 (L)			
	At1g48030 (L)			
Branched-chain alpha keto acid dehydrogenase complex	At5g09300 (E1)	Branched chain alpha keto-acids + CoA + NAD^+^ ⇔ Acyl-CoA + NADH	At1g55510: 150 kDa/39 kDa	Fujiki et al., 2000
	At1g21400 (E1)			Mooney et al., 2000
	At1g55510 (E1)		(0.95 MDa complex)	Fujiki et al., 2001
	At3g13450 (E1)			Fujiki et al., 2002
	At3g06850 (E2)			Taylor et al., 2004
	At3g13930 (E3)			Binder, 2010
	At3g17240 (E3)			
	At1g48030 (E3)			
Formate dehydrogenase	At5g14780	Formate + NAD^+^ ⇔ CO_2_ + NADH	(200 kDa complex)	Halliwell, 1974
				Colas des Francs-Small et al., 1993
				Hourton-Cabassa et al., 1998
				Jänsch et al., 1996
				Bykova et al., 2003
				Baack et al., 2003
				Olson et al., 2000
				Alekseeva et al., 2011
Methylmalonate semialdehyde dehydrogenase	At2g14170	(S)-methylmalonate-semialdehyde + coenzyme A + NAD^+^ + H_2_O ⇔ propanoyl-CoA + bicarbonate + NADH	At2g14170: 200 kDa/59 kDa	Oguchi et al., 2004
				Tanaka et al., 2005
				Kirch et al., 2004
Isovaleryl-CoA dehydrogenase	At3g45300	Isovaleryl-CoA + acceptor (ETF) ⇔ 3-methylbut-2-enoyl-CoA + reduced acceptor (ETF) (also considerable activity with other acyl-CoA's)	At3g45300: 132 kDa/46 kDa	Däschner et al., 1999
				Reinard et al., 2000
			(homodimeric complex)	Faivre-Nitschke et al., 2001
				Däschner et al., 2001
				Goetzman et al., 2005
				Araújo et al., 2010
D-2-Hydroxyglutarate dehydrogenase	At4g36400	D-2-hydroxyglutarate + acceptor (ETF) ⇔ 2-oxoglutarate + reduced acceptor (ETF)	(homodimeric complex)	Engqvist et al., 2009
				Araújo et al., 2010
				Engqvist et al., 2011
Saccharopine dehydrogenase	At5g39410	Saccharopine + NAD^+^ + H_2_O ⇔ Glutamate +-Amino adipate semialdehyde + NADH	not known	Zhu et al., 2000
				Heazlewood et al., 2003
Pyrroline-5-carboxylate dehydrogenase	At5g62530	Pyrroline-5-carboxylate + NAD^+^ ⇔ Glutamate (Glutamate-5-semialdehyde) + NADH	At5g62530: 158 kDa/59 kDa	Forlani et al., 1997
				Deuschle et al., 2001
				Deuschle et al., 2004
				Miller et al., 2009
Proline dehydrogenase	At3g30775	L-Proline ⇔ Pyrroline-5-Carboxylate	not known	Elthon and Stewart, 1981
	At5g38710			Verbruggen et al., 1996
				Kiyosue et al., 1996
				Mani et al., 2002
				Szabados and Savouré, 2010
				Funck et al., 2010
				Sharma and Verslues, 2010
				Schertl et al., in press
L-Galactono-1,4-lactone dehydrogenase	At3g47930	L-Galactono-1,4-Lactone ⇔ L-Ascorbate	(420 kDa, 470 kDa, 850 kDa complexes)	Mapson and Breslow, 1958
				Siendones et al., 1999
				Leferink et al., 2008
				Pineau et al., 2008
				Leferink et al., 2009
				Schertl et al., 2012
D-Lactate dehydrogenase	At5g06580	D-Lactate ⇔ Pyruvate	(homodimeric complex)	Bari et al., 2004
				Atlante et al., 2005
				Engqvist et al., 2009
				Wienstroer et al., 2012
Glycerol-3-phosphate dehydrogenase	At3g10370	Glycerol 3-phosphate ⇔ Dihydroxyacetonephosphate	At3g10370: 160 kDa/65 kDa	Shen et al., 2003
				Shen et al., 2006
Dihydroorotate dehydrogenase	At5g23300	Dihydroorotate ⇔ Orotate	At5g23300: 156 kDa/49 kDa	Ullrich et al., 2002
				Doremus and Jagendorf, 1985
				Miersch et al., 1987
Succinic semialdehyde dehydrogenase	At1g79440	Succinic semialdehyde ⇔ Succinate	At1g79440: 163 kDa/55 kDa	Busch and Fromm, 1999
				Bouché et al., 2003
				Kirch et al., 2004
				Toyokura et al., 2011
Histidinol dehydrogenase	At5g63890	L-histidinol + NAD^+^ ⇔ L-histidine + NADH	At5g63890: 115 kDa/51 kDa	Nagai and Scheidegger, 1991
				Ingle, 2011
Alternative NAD(P)H dehydrogenases (NDA1, NDB4, NDA2, NDB2, NDB3, NDB1, NDC1)	At1g07180	NAD(P)H + UQ ⇔ NAD(P)^+^ + UQH_2_	At2g20800: 160 kDa/65 kDa	Escobar et al., 2004
	At2g20800		At2g29990: 163 kDa/55 kDa	Rasmusson et al., 2004
	At2g29990		At4g05020: 160 kDa/65 kDa	Rasmusson et al., 2008
	At4g05020			Wulff et al., 2009
	At4g21490			Wallström et al., 2014a,b
	At4g28220			
	At5g08740			

a *Mitochondrial dehydrogenases without complex I (NADH dehydrogenase) and complex II (succinate dehydrogenase) of the respiratory chain. This list corresponds to the dehydrogenases shown in Figure 1*.

b *Accession numbers in accordance with The Arabidopsis Information Resource (TAIR)*.

c *Oligomeric state: native mass and monomer mass according to GelMap (https://gelmap.de/231)*.

d *Key publications for Arabidopsis (other plants)*.

## Electron entry pathways into the ETC

**Figure 2:**
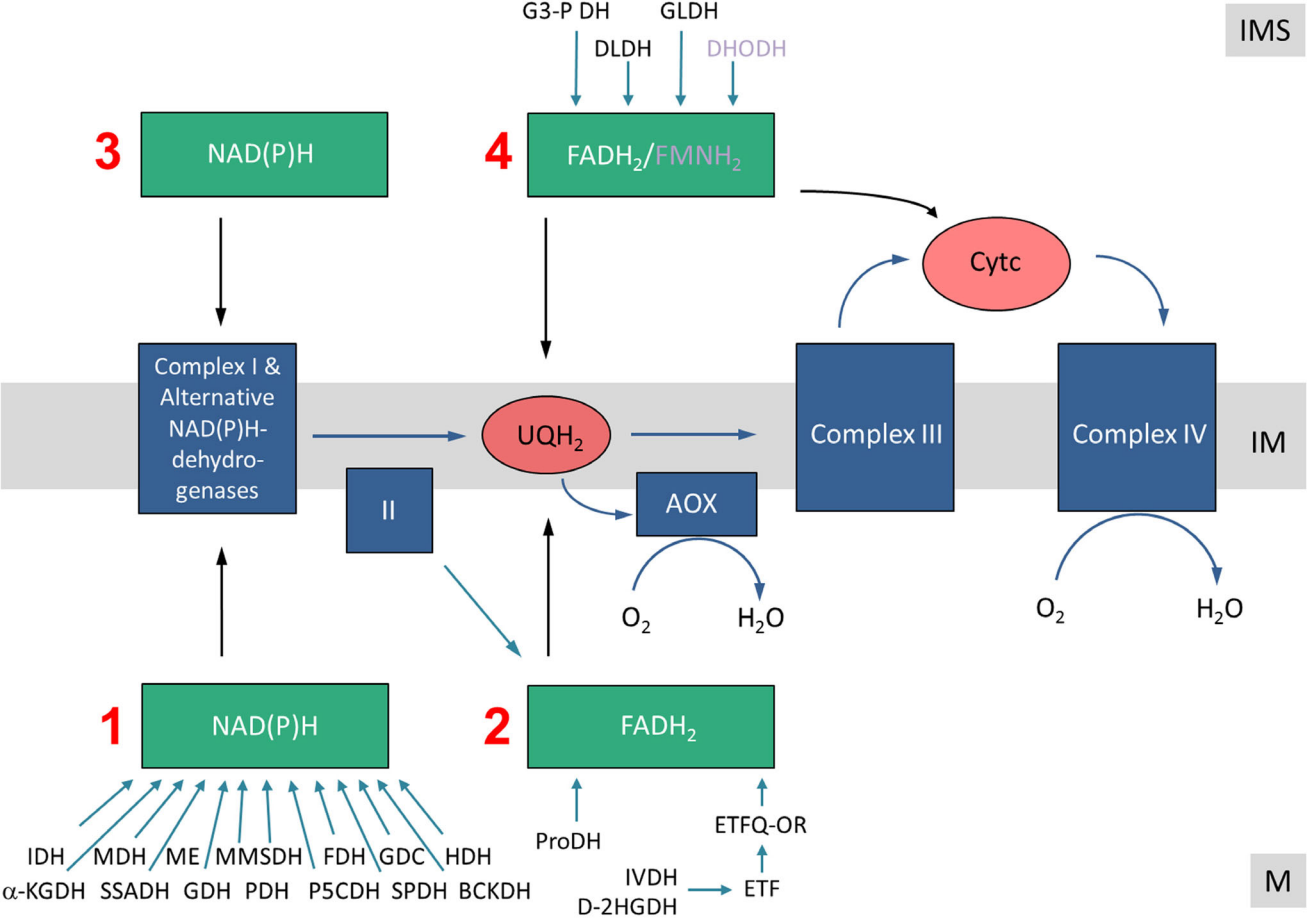
**Electron entry pathways into the mitochondrial electron transport chain in plants**. Electrons enter the respiratory chain via four different pathways. **(1)** The Matrix-NAD(P)H pathway. Various dehydrogenases oxidize carbon compounds in the mitochondrial matrix. Electrons are transferred in the form of NADH to the ETC. NADH is re-oxidized by complex I or the internal alternative NAD(P)H dehydrogenases. **(2)** The Matrix-FADH_2_ pathway. FAD-containing enzymes oxidize carbon compounds in the mitochondrial matrix and directly (ProDH?) or indirectly (via the ETF/ETFQQ system) transfer electrons to the ubiquinone pool. **(3)** The IMS-NAD(P)H pathway. Cytoplasmically formed NAD(P)H is re-oxidized via external alternative dehydrogenases. **(4)** The IMS-FADH_2_ pathway. FAD/FMN-containing enzymes oxidize carbon compounds in the mitochondrial intermembrane space. Electrons are transferred either to the ubiquinone or the cytochrome c. M, matrix; IM, inner membrane; IMS, intermembrane space. Abbreviations—*alphabetically ordered*. I, complex I; II, complex II; III, complex III; IV, complex IV; α-KGDH, α-ketoglutarte dehydrogenase; AOX, alternative oxidase; BCKDH, branched-chain α-ketoacid dehydrogenase complex; Cytc, cytochrome c; D-2HGDH, D-2-hydroxyglutarate dehydrogenase; DHODH, dihydroorotate dehydrogenase; DLDH, D-lactate dehydrogenase; ETF, electron transfer flavoprotein; ETFQOR, electron transfer flavoprotein ubiquinone oxidoreductase; FDH, formate dehydrogenase; GDC, glycine dehydrogenase; GDH, glutamate dehydrogenase; GLDH, L-galactono-1,4-lactone dehydrogenase; G3-PDH, glyceraldehyde 3-phosphate dehydrogenase; HDH, histidinol dehydrogenase; IDH, isocitrate dehydrogenase; IVDH, isovaleryl-coenzyme A dehydrogenase; MDH, malate dehydrogenase; ME, malic enzyme; MMSDH, methylmalonate-semialdehyde dehydrogenase; P5CDH, pyrroline-5-carboxylate dehydrogenase; PDH, pyruvate dehydrogenase; ProDH, proline dehydrogenase; SPDH, saccharopine dehydrogenase; SSADH, succinic semialdehyde dehydrogenase; UQH_2_, ubiquinol.

All electrons enter the ETC via NAD(P)H (generated by a variety of dehydrogenases in the mitochondrial matrix or the intermembrane space/the cytoplasm) or via flavine nucleotides (FADH_2_, FMNH_2_), which generally are bound to proteins termed flavoproteins. Consequently, the following electron entry pathways into the ETC can be defined: (i) the Matrix NAD(P)H pathway, (ii) the Matrix-FADH_2_ pathway, (iii) the Intermembrane-space-NAD(P)H pathway, and (iv) the Intermembrane-space-FADH_2_/FMDH_2_ pathway (Figure 2).

Different metabolic processes, which vary depending on the physiological state of the plant cell, contribute to the four electron entry pathways. During stable carbohydrate conditions, electrons for the respiratory chain can be supplied by NADH and FADH_2_ provided by the tricarboxylic acid (TCA) cycle. This is believed to be the standard mode of cellular respiration in non-green plant tissues or green tissues at night and resembles the basic situation in animal cells. However, during photosynthesis, NADH generation of the TCA cycle is reduced because some of its intermediates are used for anabolic reactions (reviewed in Sweetlove et al., 2010). Furthermore, the pyruvate dehydrogenase (PDH) complex is deactivated in plant mitochondria in the light by phosphorylation (Budde and Randall, 1990). At the same time photorespiration leads to an increase in NADH formation in the mitochondrial matrix by the activity of the glycine dehydrogenase complex (GDC). Indeed, at high-light conditions, NADH formed by GDC is believed to be the main substrate of the ETC, and not the NADH formed by the enzymes of the TCA cycle. At the same time, plant cells might become over-reduced in the presence of high-light. In this situation alternative oxidoreductases can insert excess electrons into the respiratory chain without contributing to the proton gradient. Upon carbon starvation conditions (e.g., extended darkness) electrons from the breakdown of amino acids are provided to the ETC (Araújo et al., 2011). Especially after release of salt stress the amino acid proline is used as an electron source (Szabados and Savouré, 2010). In summary, electron entry into the ETC is a highly flexible process in plants which much depends on light, the metabolic state of the cell as well as environmental stress factors.

## Supramolecular structure of the ETC system

The ETC is based on defined protein-protein interactions. Most stable interactions occur within the four “classical” oxidoreductase complexes of the respiratory chain. Indeed, complexes I to IV can be isolated in intact form by various biochemical and electrophoretic procedures. Furthermore, several lines of evidence indicate that complexes I, III and IV interact within the inner mitochondrial membrane forming respiratory supercomplexes (reviewed in Dudkina et al., 2008). Complex I as well as complex IV associate with dimeric complex III (I + III_2_ and IV_2_ + III_2_ supercomplexes). An even larger supercomplex includes complexes I, III_2_, and IV and was proposed to be called “respirasome” because it can autonomously catalyzes the overall ETC reaction in the presence of ubiquinone and cytochrome c. The alternative oxidoreductases of the plant ETC seem not to be part of the respiratory supercomplexes. However, alternative NDs were found to be part of other protein complexes of about 160 kDa (Klodmann et al., 2011) or 150–700 kDa (Rasmusson and Agius, 2001).

**Figure 3:**
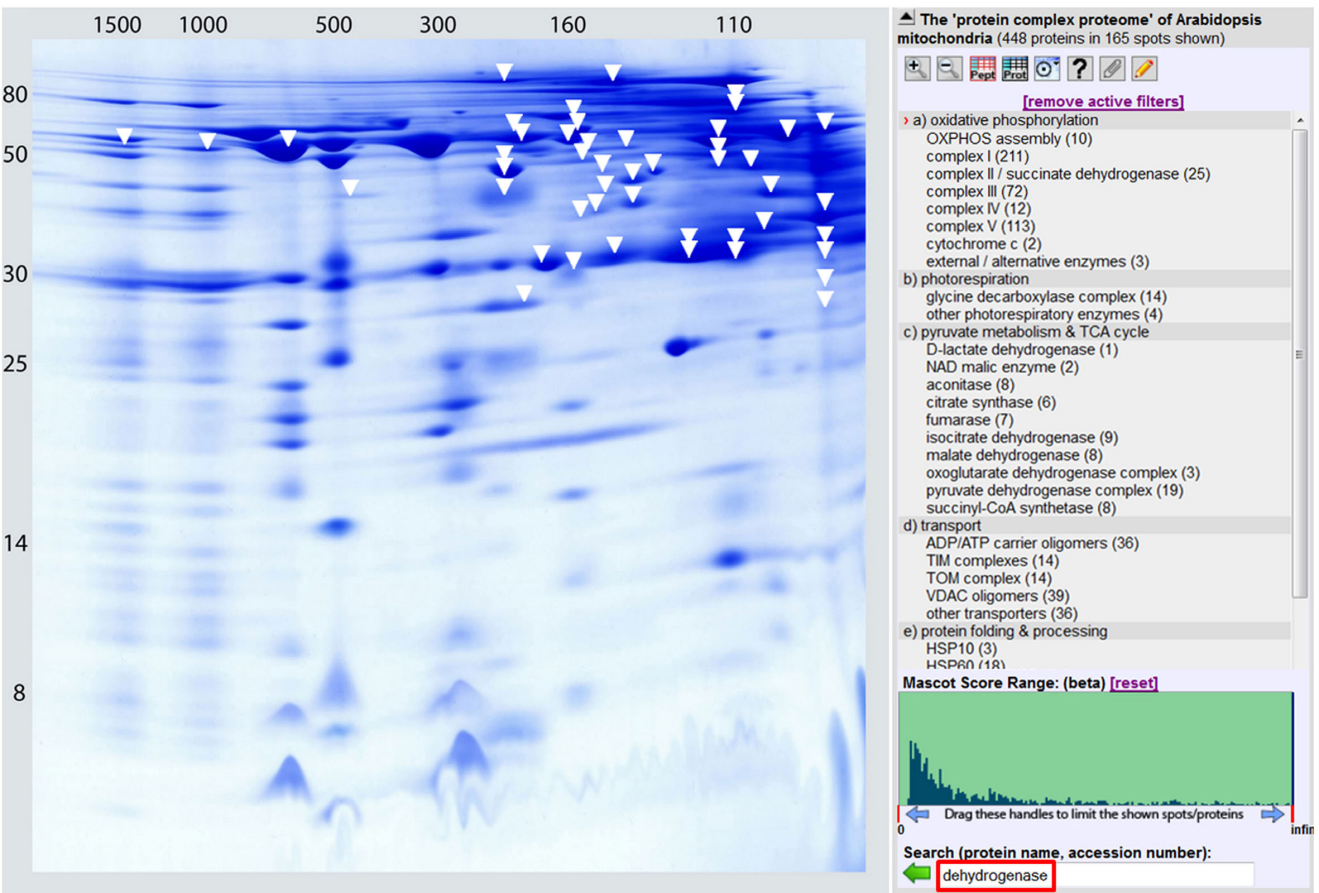
**The dehydrogenase subproteome of plant mitochondria**. Mitochondrial proteins from *Arabidopsis thaliana* were separated by 2D Blue native/SDS PAGE and displayed via GelMap (https://gelmap.de/231#). Protein separation under native condition was from left to right, protein separation in the presence of SDS from top to bottom. Molecular masses of standard proteins are given to the left/above the 2D gel. All proteins annotated as “dehydrogenase” are indicated by white arrows. Exception: The subunits of complex I (NADH dehydrogenase) and complex II (succinate dehydrogenase) are not indicated on the figure.

Experimental data also indicate that several of the mitochondrial dehydrogenases form protein complexes. TCA cycle enzymes can assemble forming multienzyme clusters (Barnes and Weitzman, 1986). In addition, some of the mitochondrial dehydrogenases interact with ETC complexes, e.g., malate dehydrogenase has been reported to interact with complex I in animal mitochondria (Fukushima et al., 1989; see Braun et al., 2014 for review). Information on the native state of mitochondrial dehydrogenases furthermore comes from the GelMap project (Klodmann et al., 2011). Using 2D Blue native/SDS PAGE and systematic protein identifications, various dehydrogenases were described (Figure 3, Table 1). Native molecular mass of the dehydrogenases in many cases much exceeds the molecular mass of the monomeric proteins (Table 1, column 3). This indicates that probably most mitochondrial dehydrogenases form part of defined higher order structures.

## Conclusion and outlook

Cellular respiration in plants is an especially dynamic system. The classical protein complexes of the ETC have extra functions and several alternative oxidoreductases occur. A network of mitochondrial dehydrogenases directly or indirectly supplies electrons for the respiratory chain. Insertion of electrons via various pathways is highly dependent on the metabolic state of the plant cell. The regulation of electron entry pathways into the respiratory chain is only partially understood and might, besides others, depend on the formation of supramolecular structures. Non-invasive experimental procedures will be necessary to physiologically investigate the function of these structures by future research.

### Conflict of interest statement

The authors declare that the research was conducted in the absence of any commercial or financial relationships that could be construed as a potential conflict of interest.
